# 

**Published:** 1852-07

**Authors:** 


Dr. Dwinelle, on Deep Seated Dental Carries, . . . . . 223

Physician Visiting List, etc., . . . . . 240

PART II.—ORIGINAL COMMUNICATIONS.

A Case of Third Dentition, etc., . . . . . 243
Letter from J. O. Martin, . . . . . 245

PART III.—EDITORIAL.

Dr. Hunter's Claims Vindicated by himself, . . . . . 247
Philadelphia College Dental Surgery, . . . . . 248
New York College Dental Surgery, . . . . . 249
Ohio College Dental Surgery, . . . . . 249
Review of Remarks on Professional Dental Education, etc., . . . . . 250
Ohio Medical College, . . . . . 250
" " and Surgical Journal, . . . . . 251
Charleston Medical Journal and Review, . . . . . 251
American Whig Review, . . . . . 251
J. D. Chevalier, . . . . . 252
New Dental Periodical, . . . . . 252
Present No. of the Register, . . . . . 253
The Meetings of Am. Society D. S., and the Mis. V. A. D. S., . . 253

